# Food Peptides for the Nutricosmetic Industry

**DOI:** 10.3390/antiox12040788

**Published:** 2023-03-23

**Authors:** Irene Dini, Andrea Mancusi

**Affiliations:** 1 Department of Pharmacy, University of Naples Federico II, Via Domenico Montesano 49, 80131 Napoli, Italy; 2 Department of Food Microbiology, Istituto Zooprofilattico Sperimentale del Mezzogiorno, Via Salute 2, 80055 Portici, Italy

**Keywords:** food antioxidant peptides, food analytical methods, large-scale biopeptide production, supplements, delivery systems, nutricosmetic, cosmeceutical, circular economy, waste recycling, anti-aging, skincare market

## Abstract

In recent years, numerous reports have described bioactive peptides (biopeptides)/hydrolysates produced from various food sources. Biopeptides are considered interesting for industrial application since they show numerous functional properties (e.g., anti-aging, antioxidant, anti-inflammatory, and antimicrobial properties) and technological properties (e.g., solubility, emulsifying, and foaming). Moreover, they have fewer side effects than synthetic drugs. Nevertheless, some challenges must be overcome before their administration via the oral route. The gastric, pancreatic, and small intestinal enzymes and acidic stomach conditions can affect their bioavailability and the levels that can reach the site of action. Some delivery systems have been studied to avoid these problems (e.g., microemulsions, liposomes, solid lipid particles). This paper summarizes the results of studies conducted on biopeptides isolated from plants, marine organisms, animals, and biowaste by-products, discusses their potential application in the nutricosmetic industry, and considers potential delivery systems that could maintain their bioactivity. Our results show that food peptides are environmentally sustainable products that can be used as antioxidant, antimicrobial, anti-aging, and anti-inflammatory agents in nutricosmetic formulations. Biopeptide production from biowaste requires expertise in analytical procedures and good manufacturing practice. It is hoped that new analytical procedures can be developed to simplify large-scale production and that the authorities adopt and regulate use of appropriate testing standards to guarantee the population’s safety.

## 1. Introduction

The cosmetic industry considers food peptides as innovative bioactive compounds for cosmetics market growth. According to the Food and Drug Administration (FDA; responsible for health products’ regulation in the USA), peptides are defined as amino acid polymers with a specific sequence and less than 40 amino acids in total [1]. According to their intended action mechanism, cosmetic peptides can be categorized into: signal peptides (which stimulate matrix protein production, cell growth, and other cell metabolic functions); carrier peptides (which help transport of active or trace elements inside the cell); neurotransmitter-inhibiting peptides (which inhibit acetylcholine release that may lead to expression wrinkles); and enzyme-inhibiting peptides (which decrease the activity of enzymes related to skin aging) [2]. Peptides have gained worldwide attention for their sustainability, with no toxic side effects [3]. The global bioactive peptide market was USD 4960.4 million in 2022 with an expected compound annual growth rate (CAGR) of 9.4% in 2022–2030 [4]. The growing number of biopeptides listed in the “European glossary of common ingredient names for use in the labeling of cosmetic products” (there were 2698 entries with the word peptide [5] in the 2022 revision compared to 848 entries in the 2019 revision [6]) demonstrates the market interest in these bioactive compounds. As a result, much research has been performed to optimize biopeptide production from natural sources (e.g., food products and protein-rich by-products of the food industries) and to examine their bioactivity in vitro (cell culture and biochemical assays) and in vivo (animal and human tests). Traditional medicine and modern scientific research consider bioactive peptides useful for formulating food supplements and cosmetic products. Bioactivity, interaction with skin cells by multiple mechanisms, high potency at a low dosage, and size compatible with penetration into the upper skin layers seem to confirm this hypothesis [7]. However, some questions remain unclear. Large-scale production, dietary interactions, and human absorption are the most significant problems to solve. This review summarizes the latest knowledge on purification and identification methods used to obtain natural peptides and the approaches used to improve their bioavailability, hoping to provide a basis for their application in the nutricosmetic market as well as a starting point for further studies.

For this purpose, systematic bibliometric analyses were performed using bibliometric records published between 1993 and 2023 in Scopus and Web of Science. These two central citation databases rank journals entries based on productivity, influence, and prestige.

## 2. Production Methods for Natural Biopeptides

**Figure 1:**
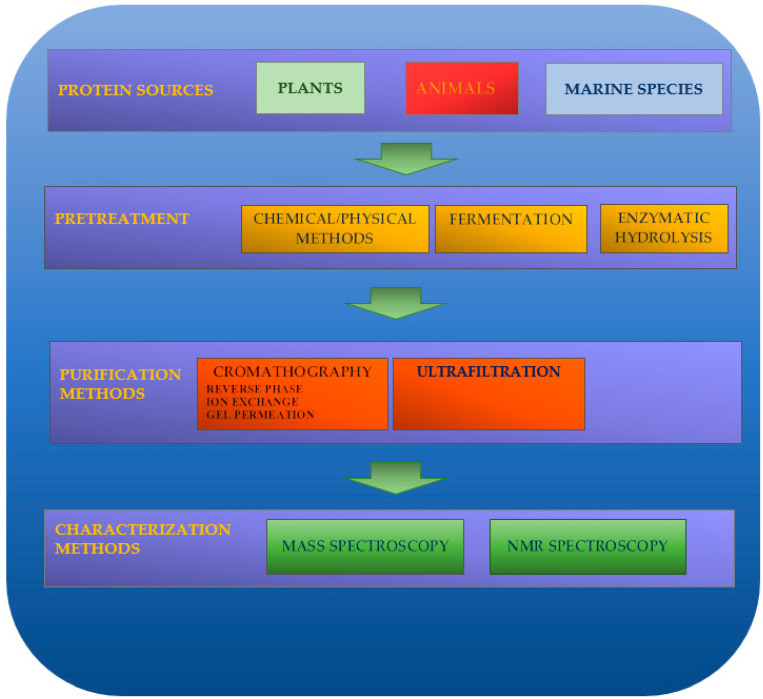
Production of natural peptides.

Natural peptides can be obtained by enzymatic hydrolysis, fermentation, and chemical-physical processes (alkaline or acidic treatments and use of microwaves, ultrasonics, hydrostatic pressure, and pulsed electric fields). Electrophoresis, membrane separation, or chromatography techniques (gel permeation chromatography, ion-exchange chromatography, reversed-phase high-performance liquid chromatography, etc.) can be used for their isolation and spectroscopic technologies (i.e., MS or NMR) as characterization techniques (Figure 1).

### 2.1. Pretreatment

#### 2.1.1. Chemical Processes

A basic or acidic environment and high temperatures hydrolyze the protein structure [8,9].

Alkaline hydrolysis is a nonspecific protein hydrolysis that cleavages amide bonds. The disadvantages of this method are the lack of specificity in the cleavage of peptide chains [10] and the loss of some amino acids (i.e., lysine, cysteine, serine, arginine, isoleucine, and threonine) [8,10,11].

**Figure 2:**
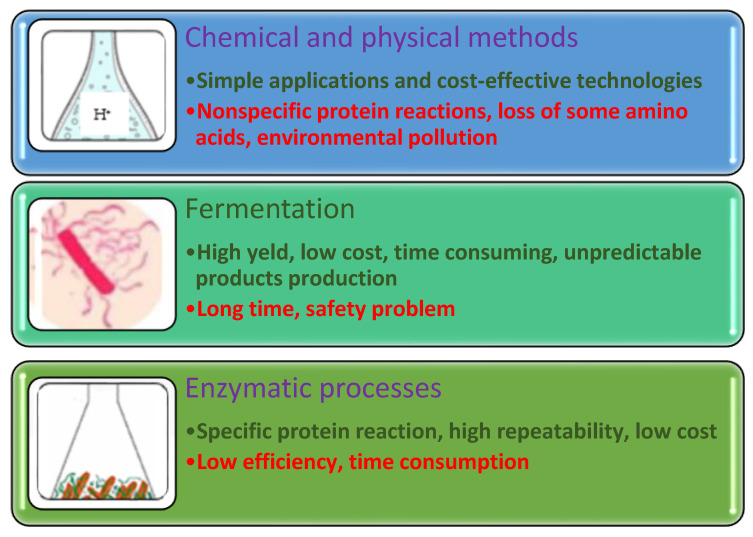
Natural source pretreatments to prepare peptides. Advantages are shown in green. Disadvantages are given in red.

Acidic hydrolysis can transform asparagine into aspartic acid and glutamine into glutamic acid and can damage tryptophan, cysteine, and methionine. The high level of salts generated by the neutralization process can also affect the antioxidant peptides’ bioactivities. Therefore, desalination is necessary before bioassay (Figure 2) [9,10,11].

#### 2.1.2. Physical Methods

The ultrasonic and microwave techniques are simple applications and cost-effective technologies used at the industrial level [8,12]. Ultrasonication systems can break molecular bonds and produce cavitational bubbles that collapse. The high temperatures, high pressure, and shock waves can damage the cell membrane and make the small molecules available [8,9]. Ultrasound increases the release of antioxidant [13] and ACE-inhibitory peptides by increasing surface hydrophobicity [14].

The microwave-assisted processing uses electromagnetic radiation (ranging from 300 MHz to 300 GHz) to extract biopeptides from protein resources. The energy is transferred through molecular interactions in the material by ionic conduction mechanisms and dipolar rotation. The extraction of peptides is due to the collision of charged ions through inter- and intra-molecular friction, resulting in thermal energy production that breaks the membranes and protein cell walls. Microwaving improves enzymatic proteolysis and hydrolysate properties (e.g., antioxidant properties), decreases hydrolysis time, alters protein conformation, and enhances enzyme accessibility [15,16].

Hydrostatic pressure uses isostatic pressures (ranging from 100 and 1000 MPa), with or without heat treatment. This technology’s advantage is minimal damage to the biopeptides due to the low temperature applied [17]. Guan et al. [18] used hydrostatic pressure (200 MPa for 4 h) to obtain biopeptides from soy protein hydrolysates with higher antioxidant activities and ACE inhibitory properties.

Pulsed electric field (PEF) technology employs short pulses of electric fields with intensity ranging from 10 and 80 kV/cm and lasting for micro-or milliseconds. PEF improves the production of biopeptides by denaturation, unfolding, or gelation. Electric field strength, pulse shape, number of pulses, and treatment time affect the process quality [14]. The use of physical methods to obtain antioxidant peptides is limited since they can lead to unpredictable reactions and force-sensitive residues (Figure 2).

#### 2.1.3. Fermentation

Microbial fermentation is an eco-friendly, cost-effective, but time-consuming method of obtaining biopeptides using bacteria or yeast; their proteases hydrolyze natural proteins into protein hydrolysates. Fermentation can make unpredictable products [19]. The microbial strain, type of protein, fermentation time, and temperature conditions can affect the yield and quality of hydrolysis [10,20]. Generally, fermentation positively impacts organoleptic and physicochemical final product quality [21] (Figure 2).

#### 2.1.4. Enzymatic Processes

Enzymatic processes use single, double, or multiple proteases sourced from plants (such as papain), from microorganisms (such as Flavourzyme, Aalcalase, and Protamex), or from animals (such as pepsin and trypsin) to cleave proteins and release bioactive peptides [22]. The enzyme/substrate ratio, temperature, pH, and hydrolysis time can affect hydrolysis [8]. For example, pepsin, trypsin/pancreatin, and α-chymotrypsin (gastrointestinal digestion enzymes) can be used to produce inhibitory peptides (SSTY-hydrolysate-derived DPP-IV) [23], and soy flour hydrolysate can be employed to produce antioxidant peptides [24]. The main advantages are control, definition, and short reaction time [19] (Figure 2). The enzyme choice must be based on the intended hydrolysate products (i.e., trypsin enzyme for hydrolyzing casein). Generally, the peptides’ activities and molecular weights decrease when the hydrolysis degree is enhanced. However, the product activity may decline when a certain amount of enzymatic hydrolysis occurs since peptides are transformed into amino acids or their active groups are destroyed. Therefore, it is necessary to strictly control the degree of enzymatic hydrolysis to ensure that the peptides formed have high activity [25,26].

### 2.2. Purification Technologies

**Figure 3:**
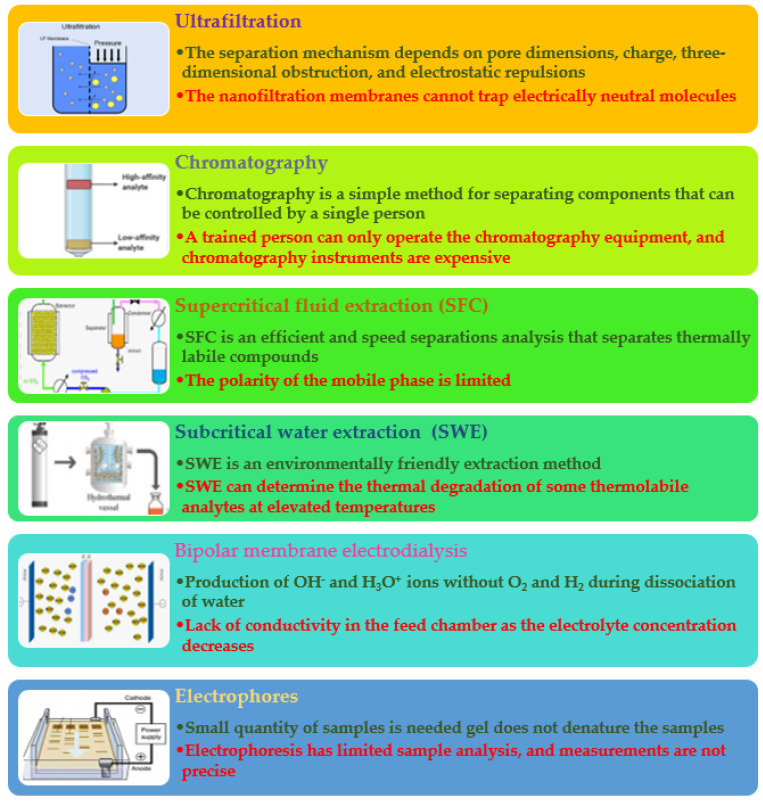
Peptide purification methods. Advantages are reported in green. Disadvantages are given in red.

The separation of peptides in protein hydrolysate can be achieved by using ultra-filtration, chromatography, supercritical fluid extraction, and electrophoresis (Figure 3).

#### 2.2.1. Ultrafiltration

Membrane separation is a technique that saves energy, does not require extra chemical agents, does not pollute, and can be combined with other techniques [12]. Permeable membranes with different molecular weights are employed to purify peptides. In recent years, microfiltration, nanofiltration, ultrafiltration, and affinity membrane filtration technologies have been developed [27].

Microfiltration is a precision filtration technique using membranes with pores between 0.1 and 1 μm. Organic (e.g., cellulose acetate, polycarbonate, polyamide, and polypropylene) and inorganic (e.g., metal and ceramic) microfiltration membranes are available commercially. Microfiltration membranes separate the proteins and peptides in the brine [28]. Ultrafiltration is a membrane filtration process employed to purify and concentrate the components of a solution using membranes with pores between 1 nm and 0.5 μm. Ultrafiltration membranes can separate molecular weight ranges between 10,000–300,000 Da.

Nanofiltration membranes contain nanoscale pores that can separate molecular weights between those intercepted in reverse osmosis and ultrafiltration techniques with a diameter of ~1 nm. The separation mechanism depends on pore dimensions, charge, three-dimensional obstruction, and electrostatic repulsions. Active peptides with similar molecular weights, but different isoelectric points, can be separated using nanofiltration. The nanofiltration membranes can trap only ions and not electrically neutral molecules [29].

#### 2.2.2. Chromatography

Chromatographic techniques are powerful tools for separating complex peptide mixtures. They are often associated with bioassay to reduce the number of peptides that coexist in the active fraction based on their chromatographic characteristics and biological potentiality [22,29]. For example, Shih et al. [30] employed cation-exchange liquid chromatography and an ACE inhibitory assay to purify biopeptides from *Cassia obtusifolia.* The chromatographic techniques most commonly used to separate biopeptides are high-speed countercurrent chromatography (CC), magnetic solid-phase extraction, and high-performance liquid chromatography (HPLC).

The high-speed countercurrent chromatography is a carrier-free liquid-liquid partitioning chromatography that needs a large amount of equipment and time. It gives high recovery rates and does not suffer from interference caused by the carrier; however, it has poor separation efficiency.

The magnetic solid-phase extraction separates and purifies compounds from a complex matrix dissolved in a liquid. A magnet adsorbs the analyte, a magnetic separator allows their recovery, and a solvent elutes the analytes. Yu et al. [31] used magnetic solid-phase extraction to purify γ-glutamyl peptides from garlic.

The HPLC is a fast separation technique that permits good separations and high recovery rates based on hydrophobic properties (reverse-phase chromatography, RP), hydrophilic properties (hydrophilic interacting liquid chromatography, HILIC), size (size exclusion chromatography, SEC), and charge (ion exchange chromatography, IEX). The HPLC can be combined with spectroscopic instruments (e.g., mass) to purify and identify peptides.

(RP)-HPLC separates peptides based on the amino acids’ hydrophobic properties. It employs a polar mobile phase and a nonpolar stationary phase. The (RP)-HPLC gives good separation, high resolution, and high recoveries. Hara et al. [32] showed that adding trifluoroethanol (10%–16%) to the mobile phase significantly increases the separation of peptides. A disadvantage of (RP)-HPLC is that it cannot be used for hydrophilic peptides [33]. The (RP)-HPLC was applied to separate wheat germ protein hydrolysates [34], cow milk products [35], turmeric and ginger [36].

Hydrophilic interacting liquid chromatography (HILIC) or normal-phase (NP) chromatography employs a hydrophobic organic mobile phase (e.g., silica stationary phases with siloxanes, silanols, and with (or without) a small number of metals, polysulfoethyl A (derivatized silica), PolyWAX (weak anion exchanger), Polycat A (weak cation exchanger), (ZIC)-HILIC (zwitterionic)), and a hydrophilic stationary phase (e.g., 70% acetonitrile, methanol or isopropanol) to separate biopeptides [37]. The HILIC technique was employed to purify biopeptides in homogenized milk [38].

Ion-exchange chromatography (IEX) separates biopeptides depending on their charge. It uses a resin that contains ionic groups (anions or cations) as a fixed phase and a polar solvent as a mobile phase. The disadvantage of this technique is the high salt concentration in the final product. A desalting step is necessary to minimize interference during peptide identification or bioassay [12].

Ion exchange chromatography is simple, resistant to alkalis and acids, and gives a high resolution. IEX was applied to separate bioactive peptides from *Boletus* mushrooms [39], sea cucumber [40], and watermelon [41].

Gel chromatography, also called size exclusion chromatography, is a fast and straightforward separation technique that uses a gel with a network of pores as a fixed phase. Solutes are eluted in order from large to small molecular size. The mobile phase viscosity must be such as to cross the column [8]. The disadvantages of this technique are low resolution, a limited peak capacity, and large eluent volumes [12]. Gel chromatography was applied to separate bioactive peptides from animal muscle proteins [42].

#### 2.2.3. Supercritical Fluid Extraction

Supercritical fluid extraction uses fresh fluid through a sample (pressure and temperature controlled) that is recovered from the extract by depressurization. This technique allows a faster extraction than traditional methods since the supercritical fluids can penetrate a porous solid more easily. Supercritical fluid can be recycled or reused, diminishing waste. Supercritical carbon dioxide (CO_2_) is used to obtain bioactive peptides from various sources (e.g., *Chenopodium quinoa*, fruit waste, *Ganoderma lucidum*) [43,44,45]. Under low-temperature conditions, the proteins cannot denature (they cannot make polypeptides and free amino acids), the amino acids cannot oxidize, and the amino acids and carbohydrates cannot generate the Maillard browning products [46]. Unfortunately, the apparatus has a high cost, solvent compression needs recirculation measures to decrease energy costs, and the modifiers used to alter the polarity of the CO_2_ require a subsequent separation process. Supercritical fluid extraction can be coupled with enzymatic hydrolysis to produce biopeptides in less time and at low cost [47].

#### 2.2.4. Subcritical Water Extraction (Pressurized Hot Water or Hot-Compressed Water)

Subcritical water employs water in a liquid state under high pressure and temperature between 100 and 374 °C. It can be used for polar and nonpolar compounds. Under these conditions, the dielectric constant reduces, the ionic product constant (Kw) increases, and the hydrogen bonds deteriorate, making subcritical water similar to methanol and ethanol (less-polar solvents). The high pressure and high temperature used separately can denature the proteins [48,49]. Subcritical water conditions (the combined effects of high pressure and temperature) break down proteins into peptides and free amino acids [50] with an irreversible first-order reaction. Hydronium and hydroxyl ion reactions confer bicatalytic characteristics on water (weak acid or base) [50]. High temperature and pressure disrupt weak interactions (e.g., hydrogen bonds) and the loss of quaternary, tertiary, and secondary structures [49,51,52]. The union of H^+^ (derived from the hydronium ion) to the N-terminal generates atom excitation and breaks the peptide bond. Finally, the OH^−^ links with the new carbon cation of the C-terminal [53]. The degradation of Maillard’s amino acids and reaction products improves the medium’s pH at temperatures above 200 °C [54,55,56]. Subcritical water was used as a hydrolysis medium to obtain protein from vegetal meal (e.g., soybean, rice bran, deoiled Oryza sativa bran) [57,58], animal sources (e.g., ice-cream wastewater, African snail *Achatina fulica*) [59] and other sources (e.g., laver *Pyropia yezoensis*) [60], obtaining hydrolysates with a strong free-radical scavenging capacity and antioxidant activity. The disadvantages of subcritical water are the lack of selectivity and cluster formation during the overheating process [61].

#### 2.2.5. Bipolar Membrane Electrodialysis

Bipolar membrane electrodialysis (EDBM) technology does not use chemicals to separate peptides. However, it employs monovalent anion exchange and cation-selective permeation membranes to separate ions and bipolar membranes that allow the production of H^+^ and OH^−^ ions from water under the current application. Mikhaylin et al. [62] used EDBM to separate casein from milk.

#### 2.2.6. Electrophores

Sodium dodecyl sulfate-polyacrylamide gel electrophoresis (SDS-PAGE) is an analytical technique for separating biopeptides based on their molecular weight. In this technique, the migration rate through the gel matrix is affected by the peptides’ size (smaller peptides migrate faster than larger peptides due to less resistance from the gel matrix), charge, and chain length. Siow et al. used SDS-PAGE to obtain peptides from *Parkia speciosa* seeds [63].

Capillary electrophoresis is a liquid-phase separation technology in which a capillary tube separates biopeptides using a high-voltage electric field as a driving force. Capillary electrophoresis can separate analytes in various separation modes (e.g., capillary zone electrophoresis, capillary isoelectric, and capillary gel electrophoresis).

Capillary zone electrophoresis separates the biopeptides based on the different charges. The speed affects the separation process [64]. The capillary isoelectric focusing electrophoresis separates analytes based on their different isoelectric points. It is generally used to separate peptide isomers [65,66,67].

Capillary gel electrophoresis separates analytes based on molecular sieving (molecular shapes and weights). It is used to separate biopeptides with many hydrophobic side chains.

### 2.3. Identification of Peptide Sequences

#### 2.3.1. Mass Spectroscopy

Mass spectrometry (MS) is a versatile analysis method for biopeptide characterization by molecular weight. MS/MS is an alternative strategy to obtain mass spectra of peptide fragment ions from a particular precursor ion. The MS can identify and quantify the peptides, ionizing them in various modes.

Electrospray ionization (ESI) employs electrical energy to transfer ions from an initial solution into a gas stage. This ionization into gas needs the dispersion of charged droplets, solvent evaporation, and expulsion of ions from the highly charged droplets [68]. ESI-MS detects femtomole quantities of multiple compounds, including non-volatile and thermolabile analytes such as peptides. It can analyze the peptides’ intact mass and amino acid sequence by MS/MS technology. The peptide bond (-CO-NH^−^) is the most common source of fragmentation. It produces y-ions (C-terminal fragment ions) and b-ions (N-terminal fragment ions). The amino acid sequence is deduced from the peptide’s fragmentation along its backbone and around the peptide bond that generates peptide fragment types. In the case of isoleucine and leucine (that have identical molecular weight), w-ions allow the characterization (the R-group is different for isoleucine (CH_2_CH_3_) and leucine (CH_3_CHCH_3_). Worsztynowicz et al. [69] used MS/MS technology to identify biopeptides from whey proteins, Karami et al. [34] from wheat germ, and Zanoni et al. from hempseed [70]. MALDI-TOF spectra analysis of peptides usually identifies only the molecular ion [M+H]+ (it is an advantage since it maximizes the signal), but gives no evidence of structure. Daughter ions formed during the application of the extraction pulse increase the background noise. This approach is poorly sensitive and selective [71]. Only highly-abundant proteins generate patterns of peptides for their unambiguous identification. The signals of co-migrating peptides are suppressed. Ayala-Niño et al. [72] studied biopeptides from amaranth seed proteins using MALDI TOF. MALDI-TOF/TOF instrumentation can be applied to obtain more specific and reliable results [73]. Cakir et al. [74] examined proteins in black cumin seeds by MALDI-TOF/TOF-MS analysis.

#### 2.3.2. NMR

The molecule momentum, in the presence of a magnetic field, can align in the same or opposite direction to the field. Two states separated by an energy gap (resonance frequency) are formed. The nucleus’ chemical environment in a molecule and the magnetic field strength affect this difference in resonance frequency [75]. The structures of 131 peptides from 17 fungi genera were unambiguously characterized using 1D and 2D NMR [76].

## 3. The Human Skin

The human skin is a complex organ with an exceptional structure. It is made up of diverse cell types and compartments with distinctive functions. The epidermis (outermost layer) contains four sublayers (strata corneum, granulosum, spinosum, and basalis) and four major cell types (melanocytes, keratinocytes, Langerhans and Merkel cells). The epidermal–dermal junction (border between the epidermis and dermis) constitutes the basement membrane (an aggregation of proteins and structures). Under the basement membrane is the underlying dermis, which contains dendritic cells, mast cells, macrophages, fibroblasts, elastic fibers, collagen, hair follicles, blood vessels, nerves, lymph vessels, and sweat glands.

The dermis allows nutrients to reach the skin and has a structural support function [77]. Aging affects all skin layers, altering their structure and function [77]. The organism’s aging is inevitably a progressive process. The consequences of aging are changes at the tissue and cellular levels. During physiological skin aging, the number of keratinocytes at the epidermis level gradually decreases. The epithelium layers atrophy. The reproductive layer cell division activity, Langerhans cell and melanocyte numbers decrease. The dermis’ connective tissue also atrophies (its cellular and extracellular matrix components diminish). The fibroblasts synthesize collagen, but the fibers are less elastic and efficient, and protein fibers, already existing, are subject to degeneration. Only the corneocyte number in the dead stratum corneum shows no changes. The accumulation of damage reduces cells’ ability to renew [78]. The decrease in lipid and CD44 glycoprotein (regulator of keratinocyte proliferation) levels, the loss of hyaluronic acid homeostasis, and the reduced cell proliferation in the basal layer contribute to this decline [79,80].

Moreover, the contact surface area between the epidermis and dermis becomes thinner, resulting in a weakened epidermis nutrition supply and a further decline in basal cell proliferation ability [81,82]. The epidermal–dermal junction and dermis also become thinner, causing wrinkle formation since there are fewer cells, with less oxygen and less nutrition. The dermal extracellular matrix (ECM) accumulates type I and type III collagens [83], and there is a decrease in the synthesis of type I/III [84], altering the elastic fiber organization [85]. The low fibroblast levels increase wrinkling and reduce elasticity [86]. Skin aging is associated with extrinsic (external) factors, e.g., UVA, UVB, temperature, environmental pollution, nutritional factors, cigarette smoke, lack of sleep, and stress [87] and with intrinsic (endogenous) factors, e.g., genetic factors, chronological time, hormones, decreased age-related antioxidant capacity, and increase in reactive oxygen species [88,89]. The principal consequences are blemished, dry, pale skin with rugged texture, visible pores, redness, small actinic keratomes, gradual loss of elasticity, and fine wrinkles [90]. The intrinsic clinical skin aging signs caused by intrinsic factors are xerosis (dry skin), fine lines, and laxity [91]. The aging signs caused by extrinsic factors are irregular pigmentation, coarse wrinkles, and lentigines (or age spots). The photo-exposed areas (e.g., the face, hands, and neck) show a more visible occurrence of these changes. The duration and intensity of exposure to environmental factors and the skin type affect the occurrence of extrinsic skin aging signs [91]. Skin aging impacts human aesthetics and increases susceptibility to infections and chronic wounds (e.g., venous, pressure, or diabetic foot ulcers, dermatitis, and melanoma) [92,93].

## 4. Biopeptides’ Potential in Cosmeceutical Applications

The increased demand for natural cosmetics has led to the formulation of a new generation of cosmetics based on active compounds obtained from natural sources such as biopeptides. Biopeptides can enhance skin health (acting against aging-related enzymes) and decrease the harmful effect of agents that produce skin injuries (acting as antioxidant, antimicrobial, and anti-inflammatory agents). Multifunctional biopeptides, which can simultaneously start, modulate, or impede multiple physiological pathways, are preferred to single-activity peptides [94]. The problems associated with using biopeptides in cosmetics concern the yields of the techniques with which biopeptides are produced (in terms of production quantity and concentration of biopeptides capable of expressing desirable bioactivity) and the biopeptides’ structural stability and bioactivity during product manufacturing and storage. Biopeptide activity is affected by pH, interactions with other components, temperature, water activity, and formulation processes (e.g., concentration, delivery of the active compounds, and packaging) [95,96].

For example, when used in gels, creams, or lotions, the parameters to consider are sensitivity to temperature and pH to guarantee the peptide bioactivity at the action site. Moreover, it is essential to realize that only the bioactive peptides with low molecular weight penetrate the skin. Biopeptides with high molecular weight, hydrophobic character, and poor aqueous solubility at high concentrations require carriers to permit their release when needed [97]. When biopeptides are administrated orally, their bioavailability (integrity during digestion, intestinal absorption, and transport) must be controlled [97] since they are exposed to gastric, pancreatic, small intestinal enzymes, and acidic stomach conditions, meaning that only minimal biopeptide levels (nano-molar or pico-molar concentrations) reach the action site [98]. Finally, some protein-derived peptides have a bitter taste. Therefore, they must be subjected to processes to debitter them and/or mask the bitter taste to enhance the sensory properties of the final product [99]. Transport systems studied to overcome these problems include liposomes, biopolymer microgel emulsions, and solid–lipid nanoparticles [100]. Some natural lipid-based systems (e.g., chitosan fabricated nanocarriers, soy lecithin-derived nanoliposome, and microgels from alginates and methacrylate) were suggested as potential inclusion complexes for biopeptides [101]. Another limitation is the risk of allergens since most peptide preparations are produced as unpurified mixtures of several components. Plant hydrolysates may contain allergens and potentially toxic contaminating compounds (environmental pollutants) [102]. Therefore, peptide preparations from plant tissue cultures (grown in the laboratory, under axenic and controlled conditions) are preferred today [103].

### 4.1. Biopeptides with Anti-Aging Properties

Some natural peptides (e.g., snake venom, yeast, skin frog, toads, spirulina, and fish) have anti-aging properties [104]. They can inhibit key physiological enzymes such as elastase, tyrosinase, collagenase, and hyaluronidase, which are involved in the degradation of the skin protein matrix and are overproduced when intrinsic or chronological aging occurs [105]. Some of these biopeptides are under patent protection, such as the pentapeptide-3 (GPRPA) (from snake venom), which decreases skin roughness and wrinkles [106], and the hexapeptide11 (FVAPFP) (from yeast) that improves skin firmness [107].

#### 4.1.1. Biopeptides That Decrease Collagenase Activity

Collagen is the most widely distributed protein in mammals. It confers support and strength to human skin and can restore flexibility and elasticity [108]. Collagen has a role in the structural integrity and strength of connective tissues (e.g., tendons, teeth, and skin) [109]. There are different forms of collagen: type I (found in the skin, bone tissues, and tendons and widely used in cosmetic formulations), type II (found in cartilage), and type III (found in vasculature and skin) [110]. The collagen-derived peptides and collagen hydrolysates positively improve skin conditions [111]. Under heat treatment, collagen is converted into water-soluble gelatin, from which can be obtained collagen peptides (by enzymolysis). Collagen peptides are antioxidant compounds that prevent dermal collagen decomposition, negatively affect collagenase and gelatinase activity, decrease skin moisture loss and reduce wrinkling; they increase skin hydration and elasticity, and address collagen degradation and elastic fiber abnormalities due to UV radiation [112]. They can improve the hyaluronic acid content in skin tissue by enhancing the expression of hyaluronic acid synthase mRNA and filaggrin and decreasing the expression of hyaluronidase mRNA. Collagen and collagen peptides can be obtained from animal tissue, poultry, livestock, fish (bones, scales, and skin), and vegetables (spirulina) [113]. The source of collagen peptides affects their anti-skin-ageing effect (Table 1) [112]. In vivo studies showed that women given oral supplementation of collagen hydrolysate showed improvements in skin hydration, elasticity, wrinkling [114], dermal thickness, firmness [115], and texture [116] and a lessening of skin pores [117].

#### 4.1.2. Biopeptides That Decrease Hyaluronidase Activity

Hyaluronic acid (HA) is an anionic, non-sulfated linear glycosaminoglycan [128]. It is a component of the dermis extracellular matrix in many human body tissues (e.g., synovial fluid, gum, eyes, heart valves, and skeletal tissues). It can maintain skin moisture (since it can bind water) [129], improve skin rejuvenation and viscosity, and decreases extracellular fluid permeability [130]. The concentration of HA in the skin naturally declines with age. The hyaluronidase enzyme degrades it producing a loss of skin strength, flexibility, and moisture. Peptides from three microalgae (*Tetraselmis suecica, Dunaliella tertiolecta*, and *Nannochloropsis* sp.) can decrease the hyaluronidase enzyme [127]. The cosmetics industry proposes products containing hyaluronic acid for topical application, for which inhibition of hyaluronic acid degradation is crucial to avoid the inflammatory process related to the exogenous application of hyaluronic acid [130].

#### 4.1.3. Biopeptides That Decrease Tyrosinase Action

Tyrosinase is a metalloenzyme (whose active site includes two copper ions) responsible for melanin (the pigment that controls skin color) production [131]. Tyrosinase overproduction causes skin hyperpigmentation, leading to a darker skin appearance (dark brown spots and irregular grey patches) [132]. Tyrosinase activity is inhibited by compounds that block the active site or chelate copper ions [131] (Table 2). High amounts of serine can bind copper [133] and affect the C-terminal tyrosine residue [134] and amino acids with hydroxyl function [133].

#### 4.1.4. Biopeptides That Decrease Elastase Action

Elastin is an extracellular matrix of highly polymerized protein that gives elasticity to connective tissues. Arteries, skin, and lungs contain elastin [138]. It maintains skin elasticity and firmness. Elastin contains two amino-acid sequences, one responsible for crosslinking and the other for hydrophobicity. The extensive crosslinking in elastin determines insolubility and durability [139]. Fibroblasts and vascular smooth muscle cells synthesize elastin until puberty and stop when the body matures. Overproduction of the enzyme elastase decreases the elastin fibers’ production [140]. Elastin-derived peptides may prevent and regulate skin photoaging (decreasing elastase activities and fibroblast apoptosis and improving the hydroxyproline content), water content, and fibroblast proliferation [141]. Aging increases elastin degradation and elastin-derived peptide (EDP) levels, enhancing the affinity and deposition of calcium [142]. Two elastin hydrolysate-derived peptides (TGVLTVM and NHIINGW) from the skipjack have shown protective effects against skin damage due to UVA irradiation through the attenuation of oxidative stress and mitochondrial damage [143]. One elastase inhibitory peptide Phe-Phe-Val-Pro-Phe (FFVPF), with significant stability in the gastric environment, was obtained from walnut meal protein hydrolysates [144]. Norzagaray-Valenzuela et al. found peptides in microalgae (*Dunaliella tertiolecta*, *Tetraselmis suecica,* and *Nannochloropsis* sp) with elastase inhibitory effects [127].

### 4.2. Biopeptides with Antioxidant Properties Derived from Foods

In the cells, oxidative stress is generated by the imbalance between the endogenous antioxidant defense system ability and free radicals, which can produce oxidants. The reactive oxygen species (ROS, e.g., hydroxyl radical (^•^OH), superoxide anion radical (O_2_^•−^), lipid radical (ROO^•^)) and reactive nitrogen species (e.g., nitrogen oxide (NO^•^)) are responsible for degenerative changes in the aging process, heart disease, arteriosclerosis, stroke, cancer, and diabetes [145]. Antioxidant molecules (synthetic and natural) decrease the risk of chronic diseases (e.g., cardiovascular pathologies, diabetes, arthritis, Alzheimers and cancer) and skin aging related to oxidative stress and control the oxidation of food nutrients [146]. Natural antioxidants are heterogeneous secondary metabolites such as phenols, vitamins (e.g., E and C), carotenoids, glutathione, biopeptides, and some enzymes such as glutathione peroxidase, superoxide dismutase, and catalase [147]. Synthetic antioxidants (i.e., propyl gallate, t-butyl hydroquinone, and butyl hydroxyanisole) have the disadvantage of high costs and potential toxicity risk [7]. Antioxidants can donate electrons, catalyze oxide-reductive reactions (e.g., antioxidant enzyme), and prevent the interaction of transition metals (e.g., copper and iron) with hydrogen peroxide and superoxide binding proteins [147]. The research on safe and high-efficiency antioxidants from natural products (especially foods) has attracted widespread attention. Vitamins, carotenoids, bioflavonoids, and peptides have attractive antioxidant potential [148,149,150].

Peptidic antioxidants (PAs) can chelate metal ions, scavenge radicals, and quench singlet oxygen. They can be ingested safely and sometimes act as antibacterial, antihypertensive, and hypocholesterolemic molecules [151]. Peptide sequences with antioxidant activity are found in food proteins and biowaste proteins, where they occur as inactive sequences. Gastrointestinal digestion, enzymatic hydrolysis, and microbial fermentation can release biopeptides from precursor proteins [152]. Biowaste peptides are attractive for industrial applications and, at the same time, promote environmental protection. The liberated peptides must be purified before determining the sequences [153,154].

Peptides with molecular weights less than 3 kDa, containing 2–20 amino acids, among which are hydrophobic amino acids (e.g., tryptophan, phenylalanine, valine, histidine, glycine, isoleucine, lysine, and proline), and having extra aromatic rings, hydrophobic properties, and donor electrons have potential antioxidant activity [155,156].

The aromatic ring guarantees that the loss of electrons will not transform the peptide into free radicals. The extra electrons can deactivate the free radicals [157]. The hydrophobic properties permit the accessible entrance of the antioxidant peptides into target organs through hydrophobic interactions with membrane lipid bilayers [158]. Nevertheless, according to Chen et al. and Tironi et al., the antioxidant capacity of peptides declines after hydrolysis [159,160].

Chen et al. [160] found that the concentration of antioxidant amino acids and the peptide sequence might affect the antioxidant potential [161]. The amino acid sequence order of antioxidant activity is Pro-Tyr-Ser-Phe-Lys > Gly-Phe-Gly-Pro-Glu-Leu > Val-Gly-Gly-Arg-Pro, when DPPH, ABTS, and OH radical assays are used to measure it [162] and the order is Trp-Pro-Pro > Gln-Pro if the hydroxyl radical scavengers are evaluated [163]. The peptides’ synergistic influence improves their antioxidant potential and avoidance of gastrointestinal proteolysis [164,165,166,167,168]. The peptides’ antioxidant properties involve free radical scavenging, metal ion chelation, singlet oxygen quenching, and lipid peroxidation inhibition (enzymatic and non-enzymatic) [169]. PAs were found in plants (corn, rapeseed, cacao seed, rice, rye, wheat, soybean, pea, and hemp seed) [170,171,172], milk [173,174,175], marine organisms (algae, mackerel, horse mackerel bonito, yellowfin, monkfish, oysters, tuna, salmon, mussel, catfish, sardine, eel, squid, and tilapia) [176,177,178], eggs (ovalbumin, yolk, and white lysozyme) [179] and animals (porcine myofibrils, skin, buffalo horn, and dry-cured ham) [180].

#### Methods Used to Evaluate the Antioxidant Potential

The most popular protocols used to test antioxidant activities employ spectrophotometric tests. They can evaluate the hydrogen atom transfer (HAT) mechanism, single electron transfer (SET) mechanism, transient metal chelation, and in the cellular system, the Nrf2/Keap1pathway.

The ORAC (Oxygen Radical Absorbance Capacity), TRAP (Total Radical Trapping Antioxidant Parameter), CBA (Crocin Bleaching Assay), and LPA (Lipid Peroxidation Assay) evaluate HAT-based reactions.

The TEAC (Trolox Equivalent Antioxidant Capacity, also known as ABTS), DPPH (2,2-diphenyl-1-picrylhydrazyl radical scavenging activity), and CRC (Copper II Reduction Capacity assay) measure the SET-based reactions [177,181].

The EECC (EDTA Equivalent Iron Chelation Capacity) and CECC (Carnosine Equivalent Iron Chelation Capacity) test the ion chelating capacity [182].

The ABTS assay’s pH strongly affects the antioxidant potential of tryptophan, tyrosine, and their derivate peptides [183].

### 4.3. Peptides with Antimicrobial Activity

The skin is constantly exposed to microbial agents. Skin aging decreases the cutaneous production of antimicrobial peptides [184]. The bioactive peptides with antimicrobial activity against *Staphylococcus aureus*, *Propionibacterium acnes*, *Pseudomonas aeruginosa*, *Enterococcus faecium*, *Acinetobacter baumannii*, *Klebsiella pneumoniae*, *Propionibacterium acnes*, and *Enterobacter species*, are promising functional ingredients in food supplements and cosmeceuticals (Table 3) [185,186,187,188]. The antimicrobial activity of biopeptides is ascribed to the formation of transmembrane channels (by polymerization or self-aggregation), which lead to cytoplasm leakage and/or cell death, and/or inhibition of cell division, protein-folding, cell wall and protein biosynthesis, nucleic acid synthesis, and lipopolysaccharide formation [189].

It seems that positive charges (ranging from +2 to +9), small size (15–50 amino acids residues), and an amphipathic structure (ca. 50% hydrophobic residues) facilitate the biopeptide’s interaction with the negatively charged membrane of some microorganisms [190].

### 4.4. Peptides with Anti-Inflammatory Activity

Inflammation is how the body restores itself after injury, replaces damaged tissue and combats pathogens [195]. Inflammation may be acute or chronic. It can remain for a few minutes to weeks or years. Inducing inflammatory process factors are lipopolysaccharide, dextran sodium sulfate, and other toxicants. Inflammatory processes induce the production of cytokines (IL1α, IL1β, IL2, IL6, IL8, IL12, TNFα, and IFNγ) by T lymphocytes cells and macrophages [196]. Moreover, inflammation involves immune systems cells such as mitogen-activated protein kinases (MAPK; intracellular serine/threonine protein kinases) [197], nuclear factor kappa B (NF-κB; which binds nucleotropic DNA and regulates the expression of inflammatory factors) [198], and phosphatidylinositol 3-kinase/protein kinase B (PI3K/Akt) [199].

The inflammation processes can contribute to aging diseases [200] and impact the pathophysiology of cancer, rheumatoid arthritis, atherosclerosis, asthma, ulcerative colitis, and type-2 diabetes [201]. Biopeptides’ anti-inflammatory effect is affected by low molecular weights (less than 1 kDa ca. 500 Da, composed of 2–6 amino acids) (Table 4) [202] and depends on the amino acid composition (number, quality, and positions). Low molecular weight peptides can reach their target place intact (they have few cleavage sites for endopeptidase enzymes) and have specific transport modes. Peptide transport modes include PepT1 transporters, cytokinesis, cellular bypass, and passive diffusion; dipeptides and tripeptides can also be in the PepT1 category [195]. Regardless of amino acid composition, highly hydrophobic biopeptides containing leucine, tryptophan, and phenylalanine have anti-inflammatory potential. Leucine and isoleucine can act on PI3K (Akt kinases in the PI3K/Akt signaling pathway) and ERK kinases (in MAPK pathways) [203]. They can mitigate the damage caused by inflammatory factors by decreasing the kinases’ phosphorylation and changing macrophages from M1 to M2 [204]. Moreover, highly hydrophobic biopeptides can avoid lipopolysaccharide (LPS)-stimulated inflammatory responses by forming peptide-lipopolysaccharide complexes and scavenging LPS through cell membrane charge exchange [205]. Positively charged amino acids (e.g., lysine, histidine, and arginine) positively influence the biopeptides’ anti-inflammatory potential, affecting the inflammatory response linked to the activation of cascade pathways and improving their absorption in the intestine [206]. Lysine can regulate the kinase ERK’s phosphorylation and the nuclear transcription factor NF-κB’s translocation in the MAPK signaling pathways [207]. Arginine can reduce p38 and ERK kinases phosphorylation (in the MAPK pathways) and decreases the expression of TLR4 receptors and transcription factor p65′s nuclear translocation (by constraining IκB kinase phosphorylation in the NF-κB pathway) [208]. The presence of glycine and glutamine also influences the anti-inflammatory activity of biopeptides. Glycine has a high affinity for calcium, interferes with Ca^2+^ signaling [209], modulates the NF-κB signaling pathway [210], downregulates inflammatory factors (TNF-α, IL-1β, IL-8, and IL-6), and modulates the MAPK pathways (JNK, ERK, and p38) [211]. Finally, the anti-inflammatory properties of biopeptides are related to the amino acid positions. Hydrophobic amino acids situated at the peptide chain’s N-terminus and charged amino acid C-terminal ends have a positive anti-inflammatory impact [195].

## 5. Peptide Delivery Systems

Chemical, physical, and biological variability can degrade biopeptides, decrease storage life, and limit their application in different formulations. Chemical instability is due to oxidation reactions, deamination, etc. Physical instability is mainly produced by aggregation, denaturation, and surface adsorption. Biological instability is due to cell enzymes, which may cause degradation or inactivation of the active molecule and loss of biological activity [217]. Using nanocarriers can enhance the biopeptide’s stability and limit side effects. Nanocarriers (e.g., liposomes, niosomes, novasomes, transferosomes, ethosomes, cubosomes, ultrasomes, photosomes, polymerosomes, nanofibres, metal nanoparticles, dendrimers, nanocrystals, carbon nanotubes, fullerene, cyclodextrin nanosponges, solid lipid nanoparticles), hydrogels, and nanoemulsions are carrier systems used for biopeptide delivery.

Liposomes are sphere-shaped vesicles with a hydrophilic core enclosed by at least one phospholipid bilayer. They can enter the skin by merging with the lipids of the stratum corneum or via the sebaceous glands [218]. The liposomes can be made with food-grade materials (biodegradable and non-toxic) and can encapsulate nonpolar, polar, and amphiphilic amino acids [219,220]. Mechanical methods (e.g., sonication, film formation, microfluidization, and extrusion), solvent replacement methods (reverse phase evaporation, injecting ethanol, and proliposome techniques), or detergent removal methods can be used to produce them [221]. The liposomes are used in lipsticks, antiperspirants, creams, deodorants, moisturizers, and hair care formulations. They are employed to improve the solubility of vitamins (e.g., A, E, and K), antioxidants (e.g., lycopene, coenzyme Q10, carotenoids, etc.), and other active biomolecules in water, facilitate the skin’s hydration and restore the skin’s epidermal layers by incorporating lipid compounds (e.g., cholesterols, and ceramides) [222]. They can deliver biopeptides in moisturizing, anti-aging creams, body sprays, deodorants, lotions, sunscreens, fragrances, shampoos, conditioning agents, etc. High production cost and osmotic stability limit their use in cosmetic products [223].

Niosomes contain one to seven bi-lipid layers, a non-ionic surfactant (spans, tweens, alkyl amides, brijs, polyoxyethylene alkyl ethers, and sorbitan esters), and an amorphous central core [224,225]. They are obtained by mixing free fatty acids, cholesterol, and a non-phospholipid surfactant.

Novasomes can deliver hydrophilic and hydrophobic molecules, have a lower production cost than liposomes [220], improve the biopeptides residence time on the dermal layers and skin penetration, decrease the horny layer barrier’s resistance and the biopeptides’ systemic absorption [226]. Novasomes have high molecule entrapment efficiency and a much lower production cost than liposomes. They have a little higher deposition volume on the skin than niosomes [227]. Moreover, they are stable at pH changes between 2 and 13 and temperatures between 0 °C and 100 °C.

Ethosomes are vesicles containing phospholipids with a high concentration of ethanol (20–50%) which improve the bioactive peptides’ permeation across the skin, mediating the disruption of the skin’s lipid layers. Ethasomes with niacinamide are used to decrease aging, pigmentation, skin blotches, and acne [228,229].

Transferosomes are deformable vesicles containing phospholipids and an edge activator (e.g., sodium chlorate, tween 80, and span 80). They can be used as curcumin, capsaicin, and resveratrol vehicles in transdermal skin layers [230], in antiwrinkle [231], and anti-aging cosmetics [232].

Cubosomes are self-assembling honeycomb-shaped liquid crystalline lipid nanoparticles (3D structures obtained from a bi-continuous cubic liquid phase with two aqueous channels divided by a surfactant bilayer) which can contain lipophilic, hydrophilic, and amphiphilic molecules [233,234]. They are used to absorb pollutants and as stabilizers for oil-in-water emulsions [230].

Ultrasomes are liposomes that contain a UV-endonuclease enzyme that repairs UV-damaged DNA and decreases the expression of pro-inflammatory cytokines [235].

Photosomes are liposomal formulations of photolyase. They are incorporated in sunscreen products [236,237].

Polymersomes are artificial vesicular systems containing block copolymers encapsulating lipophilic and/or lipophobic molecules. They have higher stability than liposomes because of their thick and rigid bilayer structure [238,239]. They enhance skin elasticity and increase the skin cells’ activation energy [240].

Biopolymer microgels are small particles comprising a cross-linked polymer molecule network [241]. They can contain natural, synthetic, or bio-polymers (e.g., chitosan, hyaluronic acid, collagen, gelatin, and polyvinyl alcohol), polyacrylamide, xanthan gum, polyethylene glycol, pectin, starch, cellulose, alginate). They can be obtained by coacervation, antisolvent precipitation, and emulsion. Unfortunately, porous microgels can diffuse small peptides. Biopolymer hydrogels are used to produce “beauty masks” [242,243].

Solid lipid particles (SLN) are a colloidal delivery system formed by crystallized lipid particles in an aqueous medium [244]. SLNs are used in cosmetic creams, lotions, and sunscreens [243].

Nanostructured lipid carriers (NLCs) are a mixture of solid and liquid lipids with a less ordered structure that load more active molecules than SLN into their pockets. NLCs are suitable carriers for volatile essential oils [245].

Nanofibers are one-dimensional nanomaterials (e.g., collagen, silk, PVP, and PVA) having a high surface area to volume ratio, high bioactive loading capacity, small diameters, and excellent absorbing capacity. They can be used for production of cleansers, face masks, and skin healing products [246].

Inorganic nanocosmetics are nanoparticles containing metals (e.g., gold, silver, aluminum, platinum, titanium) or metalloids (e.g., silica and selenium). Among metal-based nanoparticles, gold and silver are the most used. Gold has high stability and penetrability, is inert, and is non-cytotoxic. Gold nanoparticles have antioxidant and anti-aging effects, enhance skin elasticity, skin firmness, and blood circulation, and have antibacterial, antifungal, and antiseptic properties [247].

Silver has antimicrobial properties against many microbial species and is an anti-inflammatory agent. Silver nanoparticles (AgNPs) are used in lotions, skin cleansers, creams, shampoos, deodorants, and toothpaste [248].

ZnO_2_ and TiO_2_ nanoparticles are used mainly in sunscreen for UV-A and UV-B filters [249,250].

Inorganic metalloid silica and selenium are the most used in the cosmetic field. Silica has a feel-good texture and excellent penetrability and can enclose hydrophilic and hydrophobic molecules. Silicone-based vesicles are used to deliver vitamins A, C, and E and oils such as jojoba and lanolin, in emollients and creams [251,252].

Silica nanoparticles are employed in lipsticks to homogenize lipstick pigments, in anti-aging/anti-wrinkle creams, and in hair and nail cosmetic products. They can improve cosmetic products’ texture, effectiveness, and shelf-life and act as an anti-caking agent. Moreover, they have high photostability and protect against UV radiation [253].

Dendrimers are macromolecular organic nanocarriers with a network of symmetric branches (the number of branches required determines the production process) arising from a central core, with functional groups attached at their terminal ends [223]. Polyvalence, solubility, monodispersity, low cytotoxicity, self-assembling, chemical stability, and electrostatic interactions are key factors responsible for their high selectivity and precision in the biopeptides’ delivery [254]. Biodegradable polymers (e.g., polysaccharides, poly α-esters, poly alkyl cyanoacrylates, and poly amidoamine dendrimers) are used in cosmetic formulations to benefit hair (e.g., hair-styling gels and shampoos), skin (e.g., anti-acne cream) and nails (e.g., nail polishes), and as sunscreens. Dendrimers were developed to improve resveratrol and vitamins A and B6 (PAMAM dendrimer) solubility and skin infiltration [221] and give a glossy appearance to the skin and hair (carbosiloxane dendrimer able to resist oil and water) [255].

Nanocrystals are clusters of thousands of active agents linked together in a fixed pattern to form a group (sizes ranging from 10 to 400 nm) having a very high surface area to volume ratio and high solubility and bioavailability. They facilitate biopeptide absorption into the skin by creating a high biological adhesion and concentration gradient on the skin surface for long periods. They are usually utilized to administer poorly soluble active compounds [256]. Undissolved nanocrystals can aggregate in hair follicles to produce an active molecules reservoir in addition to intracellular and intercellular pathways [257,258,259].

Fullerenes (or buckyballs) are spherical structures with many carbon atoms [260]. They can deliver biopeptides in cosmetics (e.g., anti-wrinkle, anti-acne, lightening toner, pore reduction, and moisturizing creams) and sunscreen [261,262].

Cyclodextrin nanosponges are natural oligosaccharides (containing 6–8 glucopyranose molecules) with a truncated cone-shaped structure [263]. Cyclodextrin’s lipophilic cavity can encapsulate aromatic molecules, aliphatic hydrocarbons, and vitamins [264]. They are used in perfumes, tanning products, deodorants, laundry detergents, odor removers, underarm odor shields, etc. [265].

Microemulsions (diameter 10 to 100 nm) are classified as water-in-oil (W/O) and oil-in-water (O/W) based on the predominant system’s components. The W/O microemulsions are thermodynamically stable, have noninvasive administration, high solubilization capacity, and are easily formulated but require high concentrations of surfactants to stabilize them [244] and can be only employed in oral formulations that contain mainly oil (e.g., oil-filled soft capsules). Water-dispersible forms can be formulated by homogenizing the W/O microemulsion with water and a hydrophilic emulsifier to form a W/O/W type system. Mortazavi et al. used W/O microemulsion to encapsulate PKEK, a tetrapeptide that can decrease the pigmentation process [266].

The O/W microemulsion can encapsulate hydrophobic biopeptides mixed with a hydrophobic surfactant and a co-surfactant [267,268].

Water-in-oil-in-water (W/O/W) systems are used to encapsulate the water-soluble peptides. They are multicompartment liquid dispersions where the dispersed phase is an emulsion [269]. The double emulsion can mask flavor and odor and regulate bioactive ingredients released during digestion. The type of oil used significantly affects the formation and structure of multiple emulsions and the skin barrier function [270]. Their use is limited by instability [271]. The W_1_/O/W_2_ double emulsion system is a helpful delivery matrix for hydrophilic biopeptides, as shown by Ying et al. [272], who prepared applications of W_1_/O/W_2_ double emulsions containing soy peptides by a two-step emulsification process and Giroux et al. [273] who encapsulated β-lactoglobulin hydrolysate using a W_1_/O/W_2_ emulsion system, obtaining a peptides’ release inversely correlated to the oil’s viscosity and peptides’ hydrophobicity.

## 6. Conclusions

Foods and food waste are promising sources of biopeptides for the nutricosmetic industry. Using food waste for the production of biopeptides may contribute to sustainable development and represent economic advantages. Conversion of highly abundant, inexpensive and renewable biomass to obtain biopeptides for nutricosmetic formulation is dependent on purification processes and “tailor-made” manipulation of the precursor structures. This requires expertise in analytical procedures and good manufacturing practice to ensure the population’s safety. Therefore, before thinking of recovering biopeptides on a large scale from food waste, technologies are required that can produce peptides industrially as well as regulated analytical tests to ensure consumer safety.

Nanotechnology is becoming a crucial tool for developing new cosmetic and personal care products including biopeptides in their industrial formulation. Inspection of the extensive literature shows that nanomaterials that can deliver biopeptides differ in physical and chemical properties, biocompatibility, stability, site-specificity, and biopeptide-loading capability. Additional research on biopeptide applications in final products is needed to understand their potential risks and consumer acceptance.

## Figures and Tables

**Table 1:** The anti-skin-ageing effect of collagen peptides made from a natural source.

Peptides	Source	Skin-Aging Effect	Biblio
Type I collagen-derived collagen peptide Chicken collagen	Pig collagen	Enhancement of skin collagen content by changing the ratio of type I and type III collagen. No effect on skin moisturizing.	[118]
High tripeptide-containing collagen hydrolysate (HTC-col) has high tripeptides comprising the Gly-X-Y sequence.	Porcine skin	Anti-photoaging action. Skin dryness improvement.	[119]
Chicken-derived collagen peptide	Chicken collagen	Anti-inflammatory. Antioxidant. Collagen I synthesis. Improve cell proliferation on human skin fibroblasts	[120]
YGDEY (Tyr-Gly-Asp-Glu-Tyr) from.	Tilapia collagen hydrolysate	Prevention of ultraviolet (UVB)-induced damage to cells Inhibition of UVB-mediated photoaging of the skin. Improvement of the glutathione and superoxide dismutase expression. Enhancement of type I procollagen. Reduction of the ROS in keratinocytes. Prevention of DNA oxidative damage. Inhibition of the collagenase and gelatinase expression.	[121]
Ala-Tyr dipeptide	Carp skin hydrolysate	Antioxidant activity	[122]
Hydrolyzed collagen	*Prionace glauca*	Stimulation of the collagen type I mRNA by fibroblasts. mRNA production improvement.	[123]
Hydrolyzed collagen with neutrase	Alaska pollock	Antioxidant activity	[124]
Hydrolyzed collagen with pepsin under acidic conditions	*Rana chensinensis*	Antioxidant activity	[125]
Hydrolyzed collagen with pepsin, subtilisin A, and both enzymes	*Arthrospira maxima* (spirulina)	Peptides obtained from PHS showed the highest collagenase inhibition activity	[126]
Peptides	*Tetraselmis suecica Dunaliella tertiolecta*, and *Nannochloropsis*	Decrease in hyaluronidase enzyme	[127]

**Table 2:** Summary of recent studies on biopeptides that decrease tyrosinase activity.

Peptides	Source	Activity	Biblio
Skin collagen peptides (3–10 kDa fraction)	*Todarodes pacificus*	Copper-chelation	[135]
Albumin peptide obtained using papain	Rice bran	Tyrosinase inhibition, copper-chelation	[133]
HGGEGGRPY, LQPSHY, and HPTSEVY	Rice	Tyrosinase inhibition	[134]
Peptides	Faba bean (*Vicia faba*)	Tyrosinase inhibition	[136]
Water and ethanol extracts from soy milk fermented with lactic acid bacteria strains,	Soy milk	Tyrosinase inhibition	[137]

**Table 3:** Summary of recent studies on antimicrobial biopeptides.

Peptides	Source	Activity	Biblio
TITLDVEPSDTIDGVK ILVLQSNQIR ISGLIYEETR MALSSLPR ISAILPSR LPDAALNR IGNGGELPR QVHPDTGISK EAESSLTGGNGCAK	*Saccharina longicruris*	*Staphylococcus aureus*	[191]
MDN ELAAAC LRDDF GNAPGAVA ALRMSG RDRFL	*Alfalfa RuBisCo*	*Listeria innocua*	[192]
QAIIHNEKVQAHGKKVL	*Crocodylus siamensis*	*Escherichia coli*, *Staphylococcus aureus*, *Klebsiella pneumoniae* and *Pseudomonas aeruginosa*.	[193,194]
Cationic peptides	Rice bran	*Propionibacterium acnes* JCM 6473	[190]
Peptides generated by *Aspergillus oryzae*, *Aspergillus flavipes* proteases	Bovine milk	*Listeria monocytogenes* *Staphylococcus aureus* *Salmonella enterica* Enteritidis *Escherichia coli* *Pseudomonas aeruginosa*	[191]

**Table 4:** Summary of recent studies on anti-inflammatory biopeptides.

Peptides	Source	Activity	Biblio
LDAVNR (686 Da) and MMLDF (655 Da) [35]	Spirulina	IL-8 produced by endothelial cells EA.hy926	[212]
FLWGKSY	Spent hen muscle	IL-6	[200]
VLER, WVGK, VVRP, VLLF, VALVR, LFGK, FGPK	Millet bran	TNF-α, IL-1β, PGE2	[213]
DQWL	Whey	IL-1β, COX-2, and TNF-α, and the secretion of IL-1β and TNF-α proteins in LPS-induced RAW 264.7	[214]
YFVP, SGRDP, MVWGP, TGSYTEGWS	Sunflower	IL-1β	[215].
KLRSRNLLHPT, TNGRHSAKKH	Bee pollen	COX-2, IL-6, iNOS, TNF-α	[216]

## Data Availability

Not applicable.
